# An Essay on Eclampsia Parturientium, with Cases and Remarks

**Published:** 1847-04

**Authors:** John Dawson

**Affiliations:** Jamestown, Ohio


### WESTERN JOURNAL

OF

### MEDICINE AND SURGERY.

APRIL, 1847.

**Art. I,—*An Essay on Eclampsia Parturientium, with Cases and Remarks.*** By John Dawson, M.D., of Jamestown, Ohio.

Puerperal convulsions, not from the frequency of their occurrence, but from the danger connected with them, must always be regarded by the practitioner of medicine with a considerable degree of interest. Whether they are more frequent in private than in hospital practice, in villages and country situations than in cities, or had more mortality connected with them formerly than at present, are questions, the solution of which may be somewhat aided by having cases of interest occasionally reported.

Within the last eight years, we have witnessed some few cases, an account of which, from the characters of the complaint and the effects of remedies, may be of some service to the profession. We will here insert the cases, and then offer such views upon them as seem to be warranted:

*Case* 1.—Mrs. G., æt. 42 years, was taken on the 10th of December, 1843, at the seventh month of utero-gestation, with pain in the stomach, which, to use her own language, was accompanied with the sensation of a "rolling of the stomach upwards." Dr. Winans having been called in, administered anodynes and antispasmodics, which gave temporary relief.

Dec. 11, 2 o'clock, A. M. I accompanied Dr. W. at this visit, and found the patient just recovering from a convulsion, the principal features of which denoted it to be of the apoplectic kind ; the system was violently agitated and the countenance very much distorted. Bled ℥xxx.

At 6 o'clock, we were summoned again, with the news that she was laboring under another convulsion. It being now about daylight, the countenance was found pale and distorted; intellectual faculties deranged; pulse frequent and rather feeble; stertor; and froth working out of the angles of the mouth.

On examining the arm to bleed her again, we found that the orifice previously made had come open in her struggling, and that she had bled ten or twelve ounces. Ordered a large dose of cream of tartar and jalap.

At 2 o'clock, P. M., visited her again, and found that since our last visit she had had three convulsions, and was at the time laboring under deep coma, full and hard pulse, with a wild expression of the countenance and stertorous breathing. Bled ℥xl. Shortly after this, she became sick; perspiration made its appearance, and after this she seemed more tranquil.

At 6 o'clock, P. M., saw her again; and finding that there had been no convulsions since the last visit, she was examined per vaginam, and the os tincæ found opened to about the size of a quarter of a dollar, but rather rigid.

We now undertook to ascertain if the fœtus was still living. The stethoscope was applied over the region of the uterus ; but neither the pulsations of the fœtal heart nor the bellows sound of the placenta could be heard. Besides, there was an escape of gas of an ammoniacal odor from the vagina, which Dr. Winans said that, in an experience of some twenty years, he had observed to coincide with the existence of a dead fœtus *in utero.*

Feeling satisfied that the fœtus was dead, secale cornutum was administered at proper intervals, in two scruple doses, until two drachms were taken. No effects followed, not even the slightest uneasiness in the uterine region.

On the 12th, an examination per vaginam was again made. The os tincæ was now found sufficiently dilated to accomplish the delivery, which was done, of a dead fœtus.

On the 13th, the patient had two motions of the bowels, from the effects of the cream of tartar and jalap previously administered, and from this period had a rapid convalescence.

*Case* 2.—Mrs. H. S., a lady about 20 years old and apparently of good constitution, was taken, during the seventh month of utero-gestation, with pain in the stomach, after eating some green, sour apples. She had, however, previously experienced some slight pain in the head.

She was first visited at 2 o'clock, A. M., on the 12th of September, 1844, and was found complaining of pain in the stomach alternating with pain in the head, with occasionally an effort to vomit; pulse somewhat accelerated and small; face once in a while flushed and then pale; skin slightly exalted in temperature; tongue clean; respiration natural. Bled ℥xvi, and gave an antispasmodic of sulphuric ether and laudanum. This prescription having done but little or no good, an emetic of ipecacuanha was administered, which produced free emesis and relieved the pain in the stomach.

At 7 o'clock, same date, she was still complaining of severe headache, with inability to keep her eyes fixed on any one object; skin hot; face flushed; eyes suffused; pupils rather contracted. Prescribed the cold douche to the head, and a cathartic.

In about ten minutes after taking the cathartic, a convulsion made its appearance, and continued about two or three minutes; a second followed in about twenty minutes; a third soon made its appearance; and before they were arrested, she had thirty-five paroxysms of the same character, with intervals of some fifteen or twenty minutes between them. After the eighth, an examination per vaginam was made. The os tincæ

was found to be slightly dilated, sufficient to ascertain that there was no pulsation in the umbilical cord. A dilatation was then attempted and perfected sufficiently to use the crotchet, by which the fœtus was extracted.

The convulsions continued after the delivery of the fœtus, until, as above remarked, she had in all some thirty-five. The countenance during these fits became very pale and haggard; the extremities cold; the pulse small and irregular; the respiration very laborious, and the ability to swallow almost entirely suspended.

In this stage of the case, a dose of sulphuric ether and laudanum was with some difficulty introduced into the stomach, and an application of oil of turpentine, tincture of capsicum and mustard made to the extremities, for the purpose of producing prompt vesication. The effect of this application was very salutary. The extremities soon grew warm, the circulation became equalized, and the convulsions gradually diminished in frequency and severity until they entirely subsided. Her convalescence was slow, and denoted that the brain had been dangerously assailed.

*Case* 3.—Mrs. J., æt. about 20 years, of good constitution, began to complain, during the sixth month of utero-gestation, with pain in the head alternating with pain in the stomach. This case occurred about the first of April last.

Dr. Winans was immediately called in, and bled her, after which he administered an active cathartic. In about an hour after Dr. Winans's visit, I saw the patient in consultation with him. At this time she was laboring under a convulsion which had followed a paroxysm of vomiting. After the convulsion subsided, she was again bled; but owing to the smallness of the vein and the weakness of the pulse, we did not succeed in getting a sufficient quantity of blood. It was, however, but a few minutes after the second, until a third convulsion followed; and they continued to recur, with intervals of twenty or thirty minutes, until she had some eight or ten.

An examination per vaginam was made, but the os tincæ

was found completely closed up and rigid. Another attempt was made to draw blood, which was successful. During the course of the day, the uterus was examined with the stethoscope and per vaginam, to ascertain the condition of its contents; but neither the pulsations of the fœtal heart, the bellows sound of the placenta, nor any other test that could be relied upon, gave us any evidence that the child was alive. Besides the absence of the usual signs of life, the fœtus *in utero* could be moved from side to side of the abdomen without imparting to the hand the least evidence that it was still living.

The convulsions being still urgent, and the evidence being pretty conclusive that they could not be arrested until the contents of the uterus were in some way or other removed, a dilatation of the uterus was attempted, and, by the advice of Dr. Joshua Martin, the perforator was used in order to expedite as much as possible the delivery. We did not, however, succeed; for after opening the head of the fœtus, it was found impossible to extract it. Some uterine pain having been developed during our operations, it was thought prudent to cease all mechanical appliances, and trust for a time to the pains of nature for its expulsion. We were not disappointed: the uterus rallied its energies, and expelled the fœtus in the course of some three hours.

On the third or fourth day after the fœtus was delivered, puerperal mania made its appearance. But as I intend to make this form of puerperal disease the subject of a future notice, any further remarks on the case in this connection will be omitted.

*Case* 4.—Mrs. Q., a lady of feeble constitution, æt. 38 years, and the mother of eight children, was afflicted with intermittent fever about three weeks previous to giving birth to a healthy child. The accouchement was as favorable as usual, and took place on the 20th of September, 1845. A few days after her confinement, she commenced complaining of a periodic pain above the right eye, which grew worse every succeeding paroxysm. On the 9th of October, her husband procured a box of patent purgative pills, three of which operated to excess, producing hypercatharsis. While the pills were operating, she complained of dizziness, and said she feared she should go crazy. Shortly after this, her mind became confused; she lost the ability to recognise her friends, and, to some extent, the power to speak.

At 5 o'clock of the above date, (October 9,) Dr. Winans saw her for the first time. She was then breathing slow and hard; eyes turned to the right side, at times fixed, then quivering in their sockets; pulse slow and soft; skin moist; right arm and leg partially paralyzed; deglutition difficult; and the power to speak entirely gone. On raising her head off the pillow, she was seized with a spasm. Prescription, tinct. opii gtt. xx, and a blister to the back of the neck; tinct. opii to be repeated if spasms returned.

On October 10th, A. M., she was visited again, and the friends reported that through the past night the spasms had recurred. She was now comatose. Prescription, cream of tartar and jalap. At 2 o'clock, P. M., the medicine operated on the bowels; but later in the evening, the spasms changed into hard convulsions, and at 11 o'clock; P. M., she was found to be dying.

*Case* 5.—Mrs. L., a lady of apparently good constitution, was taken, in the month of April, 1840, at the last period of her second pregnancy, with pain in the head and stomach. When she was first seen, she was walking the floor, crying out, "Oh, my head! oh, my stomach!" She was bled freely; after which the uterus was found to be contracting vigorously; and an examination being made per vaginam, the head of the child was found to have descended into the lower strait. Anticipating convulsions, she was immediately delivered with the forceps. Before the delivery was accomplished, she was seized with a violent convulsion, which continued until after the placenta was removed. After the convulsion went off, she lay in a profound coma, which continued for about an hour. She then partially returned to her senses, and answered some questions indistinctly, her tongue being somewhat paralyzed. In less than three hours after delivery, a second convulsion followed, which again left her in a state of coma. From this condition she never recovered; a third soon followed, in which she expired.

The child of this woman was delivered alive, and, to all appearances, in a healthy condition.

*Case* 6.—Miss Q., an umarried female, was taken in labor in 1839. A midwife was called in to manage the accouchement. During the progress of the labor, she was seized with a convulsion. A physician of the vicinity was called, who used the perforator; and after some considerable difficulty, with the assistance of Dr. Winans, the delivery of the fœtus was accomplished. The convulsions after delivery ceased for about an hour or more, after which they returned, and the patient expired.

In this case, the pulse was small, and the system in a state of prostration. All attention, therefore, was directed to the delivery, in the hope that when this was accomplished, the convulsions would cease. In this there was a disappointment. The system never rallied; the pulse contined small and weak until the patient expired.

*Case* 7.—Mrs. K., during her third pregnancy, and at the seventh or eighth month of gestation, was attacked with convulsions. She had suffered eight paroxysms before anything was done for her relief. After the eighth fit passed off, she was bled freely, and took an antispasmodic. This arrested the convulsions, leaving the patient comatose. The uterus was now examined, and found to contain a dead fœtus. This was removed, leaving the patient still in a state of coma.

Some two or three days after she was delivered, she was attacked again with convulsions. She was again bled, also blistered and purged. This last attack gave rise to hemiplegia and idiocy, which have resisted everything addressed for their removal. Since the advent of these lesions, she has conceived three times. In two of the instances, the children were premature; the other one went to full time, and was delivered in a healthy condition.

During the accouchement, it was noticed that only one-half of the uterus contracted, the other half being paralyzed in the same way as the extremities of that side.

---

REMARKS.

The grave character of the malady, of which the cases detailed are perhaps a fair sample as it occurs in country practice, makes everything connected in any way with its origin, pathology, or treatment, a matter of serious interest to the physician; for as we have seen, it at times makes its advent and accomplishes its fatal work without giving much time to reflect upon its causes, or the treatment proper to be instituted.

Concerning the habit of those most predisposed but little is certainly known. Among the cases detailed are all the principal varieties of habit—the weak and effeminate, the vigorous and robust. Authors, however, in regard to this matter, think that irritable and plethoric women are most liable to the epileptic and apoplectic varieties, while those laboring under a nervous diathesis are oftener the subjects of the species generally denominated the hysterical. Still, as before remarked, our knowledge in relation to this part of our subject is limited. We often see the extremes of habit exempt, while those not at all remarkable for any peculiarity in this respect are the subjects of an attack.

All experience seems to warrant the conclusion that women are most liable to convulsions during their first labor. Of the seven cases detailed in this paper, three took the complaint the first time they were confined in childbed, two at the second time, one at the third, and the other at the ninth. Dr. Clark reports nineteen cases, sixteen of which happened to women at their first pregnancy; Merriman forty-eight, thirty-six of which were at first pregnancy; Collins thirty, twenty-nine of which were at first pregnancy; Lee fifty-four, twenty-six of which were at first pregnancy, three at the second, two at the third, two at the fourth, one at the ninth, one at the tenth, and of the remaining nineteen cases there is no account.

Reasonably, from these facts, it may be inferred that some of the things pertaining to the etiology of this malady may be most successfully sought for by an investigation of the circumstances connected with this period. What is there, then, in a first more than any other pregnancy, which predisposes females to an attack of the malady? To this question, in the present state of our knowledge, it is hard to give anything like a satisfactory answer. As a general principle it may be submitted, that the changes made on the constitution of females by conception and pregnancy are by no means prejudicial to health. Married, always enjoy better health, *cœteris paribus*, than unmarried females. While, nevertheless, the validity of this position cannot be assailed, it is equally true that many females, in undergoing the changes incident to pregnancy, become affected in ways that predispose them not only to convulsions, but also to other complaints of both mind and body. How often is it the case that we see a woman of happy disposition, dignified address, and well balanced mind, rendered peevish, fretful, irritable, and, in some instances, imbecile, from the alterations made on her system by pregnancy!

Comparatively speaking, it is also a fact that very few women approach the period of parturition the first time without some considerable degree of apprehension. In part, this proceeds from the dread of what in reality they have to suffer; and in very many cases this dread is increased by reports of accidents and fatal cases unguardedly made in their presence. From data of this character they augur for themselves the worst of consequences in their approaching confinement; and it too often happens that unless great care is exercised in expelling these illusions, and inspiring a reasonable degree of hope and confidence in the ability of the attending accoucheur, their apprehensions are realized.

In the organism, also, changes are frequently developed during a first pregnancy that exercise an unfavorable inflence. We allude to those which take place primarily in the organs of generation, and secondarily in other parts, between which and these there exists a close sympathetic relation. The distension of the abdomen, enlargement of the walls of the uterus, and alterations in the nutritive functions incident to pregnancy, are more severely felt when the system is for the first time undergoing these changes than at subsequent periods. To the febrile disposition occasionally attending pregnancy, to indigestion, costiveness, and cerebral disturbances, they, it is reasonable to suppose, are at this period most liable. Enumerated also in connection with these circumstances, any one of which it might be presumed would be adequate to predispose the system to convulsions, the changes which take place in the blood, as all know, impart to the system more or less of an inflammatory diathesis, which undoubtedly would be more seriously felt in a first pregnancy than afterwards.

Enough, we conclude, has been submitted in our attempt to account for the greater liability of women in first than in subsequent labors; we will therefore proceed to another part of our subject—the period of gestation in which convulsions are most liable to occur:

By reference to the notes of the cases we have detailed, we find that the disease occurred to one at the sixth month of gestation, to two at the seventh, to one at the eighth, to one at what was supposed to be the proper period of parturition, to one some twenty days after being delivered of a healthy child, and to one both before and after being delivered. In Dewees's System of Midwifery there are some eight cases of convulsions detailed; of these it may be inferred from what is said, that the disease happened to three during the eighth month of gestation, and to the other five some time in the ninth or at the proper period of parturition. Dr. Lee remarks: "They may occur in the latter months of pregnancy, before the uterus has begun to contract, during the different stages of labor, and several days or weeks after delivery." He adds: "I have never met with a case of true puerperal convulsions before the sixth month of pregnancy."

From all these facts, it appears that the complaint may take place at any time during gestation subsequent to the sixth month, at the proper period of parturition, or after the birth of the child. It seems, however, to be least common during the early months of pregnancy and after the delivery of the child.

That convulsions, like some other puerperal affections, are more prevalent during some seasons than others is a position not supported by anything in our limited experience. Three of the cases detailed occurred in one year in the practice of myself and partner, Dr. Winans, and these happened, too, at a time when other puerperal diseases were common. Still we regard this as a mere coincidence, not entitled to any confidence in making up an opinion on the subject. It might be remarked here, that Denman thinks women more liable some years than others.

Most writers from the time of Burns make distinctions in the disease, dividing it into some three species, the hysterical, the epileptic, and the apoplectic. Of the propriety of these distinctions we suppose there is no doubt. Keeping up a distinction between the hysterical and other kinds is indispensable, from the fact that the therapeutics proper and efficacious in the former may prove detrimental if not fatal in the latter. Burns says: "The most frequent species of puerperal convulsions is of the epileptic kind, which occurs fifty times for once that the others appear." Seven of the cases reported by Dewees were either of the epileptic or apoplectic variety, the other one being of a hysterical character. The cases we have detailed were of the epileptic or apoplectic kind. Women of different constitutions and temperaments it might be presumed would be liable to different species of the complaint. With those of weak, effeminate powers of constitution and nervous diathesis, subject to occasional attacks of hysteria, the hysterical variety would be most common. On the contrary, women with a full habit of body, irritable in disposition, or having labored under some disease of the brain or epilepsy in early life, would be most likely to have the other varieties.

*The Signs which Precede and Attend Convulsions.*—With respect to the signs which precede convulsions, it may be stated that when they are appreciable at all, they vary according to the species of the complaint with which the patient is affected. Those attending the hysterical variety have of course more or less of what are usually denominated hysterical symptoms. According to Burns, the fits are preceded by a timid state of the mind, are of sudden invasion, attended with globus or struggling about the throat, have no foaming of the mouth, nor much distortion in the muscles of the face. Dewees regards opisthotonos as strongly marking this species of convulsion. The circulation is not so much disturbed in this as in the other varieties, the pulse never acquiring that extreme degree of velocity and tenuity.

Denoting the approach of the epileptic and apoplectic varieties are a number of signs so generally common to both, that for all practical purposes they may be considered together. In both varieties, cerebral disturbances are predominant. For days, sometimes, before the accession of the fit, there is a sense of fullness in the head, swimming, vertigo or pain; and often patients complain of a want of steadiness in the faculties of the mind. Occasionally the convulsion is ushered in by intense pain in the head: the patient cries out, "Oh, my head! my head!" In some instances, the pain is confined to a particular spot, the patient describing the sensation as being that of a nail driven through the head. Associated with this distress in the head, there is in some cases a violent cramp or pain in the stomach. This phenomenon has been noticed by Burns, Denman, and Dewees: the last, however, says that he knows nothing of it from his own experience. In four of the seven cases reported in this paper, the women complained more or less of distress in the stomach. In the first case, the sensation was that of a "rolling of the stomach upwards;" the second case was ushered in with pain in the stomach, after eating green, sour apples; in the third case, the patient complained alternately of pain in the head and stomach; when called to the fifth case, the woman was found walking the room and crying out, "Oh, my head! oh, my stomach!" During the presence of the fit, there is often manifested a phenomenon that appears to be characteristic. The following is Denman's description of it: "With the foaming of the mouth, there is also a sharp hissing noise, produced by fixing the teeth and by the sudden motion of the under lip, as if attempts were made to retract the saliva back into the mouth; and by this noise I have been generally able to discern the state of a patient in convulsions, though she was in another room." It is most likely that Dewees refers to this same thing, in speaking of "a peculiar sibilating noise made by the mouth, not unlike what is termed a cat spitting." In no case did we witness the *aura epileptica* so common in epilepsy.

Cause.—On the etiology of this malady it is difficult to write with precision, owing to the fact that as a general rule, this, like other complaints involving the integrity of the nervous centres, is imperfectly understood. Occurring in opposite conditions of the general system, and proceeding at times from causes altogether different in their nature, the complaint is often the result of lesions, functional or structural, not easily comprehended. Altogether the most formidable in its character in every case is the trouble in the cerebral organs; indeed, this seems to be the *sine qua non* of the disorder. But whether this proceeds from primary disease of the brain and its appendages, or from a source of irritation located in some other part of the system, by which the brain becomes sympathetically affected, are questions of perhaps as much importance as any that we are called upon to decide.

That the primary cause is often in the brain seems to us quite probable. This may proceed, as we suppose, from mental distress, or from there being a habitual determination of blood, during gestation, to the head. In regard to the former, it is said that it has been more particularly observed among those women whose unfortunate situation renders pregnancy an evil instead of a blessing; for from their seclusion, and from a deprivation of the comforts of society, their sense of present ill or apprehension of future distress, such women are especially subject to convulsions at the time of labor, and to become maniacal after their delivery. (Denman.) Sudden and violent impressions on the mind are, indeed, if we credit the best of testimony, capable of inducing an attack of convulsions, without the concurrence of any other appreciable cause. With respect to cases, also, originating from hyperemia of the vessels proper of the encephalon, we think there can be but little doubt. A female laboring under a tendency to headache or any vertiginous complaint previous to pregnancy, or to epilepsy in early life, would be most likely, as gestation progressed, from the pressure of the child on the abdominal aorta, to have such an affection increased; and this, if sufficient to produce compression of the brain, would end in convulsions. Besides, we know that in many instances the disease of the brain is such as to give rise to lesions producing hemiplegia, idiocy, and occasionally sudden death. The examples there have been of serious and fatal lesions of the brain, although of nothing like an identical or constant character, are sufficient to make the position very plausible, that the nervous centres, in some cases at least, are primarily assailed. While, however, we conclude that this is the case, we are aware that by far the largest proportion owe their origin to sources of irritation located in other parts of the system.

Great distension of the uterine fibres, or rather the alterations the parietes of the uterus undergo in the last period of gestation, may be reckoned as exercising some agency in the production of the malady. This may be inferred, among other things, from the fact that convulsions are much more common, as we have seen, in a first than in subsequent labors. It seems also that they are most frequent at the latter periods of gestation, the time when the uterine fibres, as Dewees remarks, are at their greatest stretch. These alterations in the uterus, when associated with other things favorable to convulsions, may be regarded as making up a condition of the system in which, relatively speaking, the complaint is not unfrequently developed.

Another agency occasionally found in operation, is that proceeding from a dead fœtus *in utero.* In his work on midwifery, Denman says: "When women have convulsions, the death of a child ought generallly to be esteemed rather an effect than a cause." Generally speaking, this is probably correct; still there are cases in which a dead child in the uterus seems to be the only appreciable cause, giving rise to a kind of uterine irritation that is ultimately manifested in convulsions. After the death of a fœtus *in utero* from accident, violence, or anything of the kind, all the blood which it received must necessarily be retained in the vessels of the mother. The impulse imparted to the circulating fluid of the mother by this change would of course exercise no favorable influence in a female otherwise predisposed, and probably would be adequate to produce the malady. What also to some extent favors this position, is that very commonly, after a woman has been delivered of a putrid child, the convulsions subside.

Irregularities of diet, indigestible food, or the excessive use of stimulants, there are good reasons to believe, occasion once in a while the malady before us. In one of the cases we have detailed, the woman had eaten a quantity of unripe, sour apples, and shortly afterwards commenced complaining of her stomach and head. Some of the cases related by Dr. Lee also followed the use of indigestible food and stimulants. Denman relates a case which fell into a convulsion from the presence of some offensive matter in the stomach. After the first fit, he advised the woman to irritate her throat with her finger until she vomited five or six times, after which the fits subsided. The severe pain in the stomach, of which some patients complain, may have some connection with the ingesta. Of this, however, we know nothing from experience, except of the case related, in which, to say the least of it, there was a coincidence: the patient partook of the indigestible foood, and the act was followed by pain in the stomach and convulsions.*

* In January, 1842, Dr. Winans and myself were called on to attend the accouchement of a negress pregnant with her second child. She had gone to full time, and was complaining of irregular pain in the uterus, that worked up into her

As all know, during pregnancy the appetite is subject to great changes. Anorexia there is sometimes, but more commonly a craving desire for food in large quantities and of objectionable qualities, affects many women during the last months of pregnancy. Indulged in, we can see no good reasons why these indigestible articles may not irritate the alimentary canal so as to give rise to convulsions in pregnant women, where the predisposition is already considerable, in the same way that they cause them in children and other classes of persons laboring under great gastro-intestinal sensibility. Hence it is always proper to enjoin caution with respect to this matter on those women who labor under the depraved state of appetite to which we have alluded.

Differing widely from the conditions of the system to which we have adverted, there is another in which the disease has been manifested, that will next claim attention. This seems to be the consequence of great exhaustion from hemorrhage. Burns notices convulsions originating from this cause, and submits his opinion on the treatment proper to be adopted. In the number of the Western Journal for July, August and September, 1836, there is an extract from the Southern Medical and Surgical Journal, in which is contained an account of two cases that the writer attributed to hemorrhage from a relaxed and flaccid condition of the uterus. In one case the hemorrhage preceded, in the other it succeeded delivery; in

---

stomach, giving rise to excessive cramping sensations in this organ, with occasional vomitings. Laudanum in large and repeated doses was administered without producing any relief. The os uteri was examined, and found to be dilating. She continued in a lingering condition for some hours, during which time the pain in the stomach was periodical, corresponding to the labor pains, and intensely severe, so much so that at each time they recurred, she screamed violently, and threw herself in all possible attitudes to get ease. After being in this condition for some length of time, and finding no relief from anything done, she took a notion for whisky. Some being obtained, she drank about one pint without the knowledge of any one but herself. The effect of this was prompt relief, without producing intoxication. The pains also after this became regular, and she was speedily delivered. This woman said that her first labor was characterized in a similar manner.

both, the convulsions subsided on the use of frictions to the hypogastric region, continued until the uterus was felt to have contracted.

Besides the causes to which allusion has been made, this malady is apparently at times the result of constipation, of a peculiar condition of the nervous system of the uterus, or of the pains of labor. A loaded state of the larger bowels would as a matter of course destroy the equilibrium of the circulating fluid, by lessening the quantity to the inferior extremities, and increasing it to the superior and the cavity of the cranium. As the result of this, abnormal accumulations in the vessels of the brain might arise sufficient to cause compression or some o her derangement of that organ. That the complaint is occasionally also the consequence of a peculiar condition of the nervous system of the uterus, there is good testimony. This may be an unnatural degree of irritability in the uterus, or rather in the nerves of this organ. How often, as previously remarked, is it the case that women, after impregnation, are rendered peculiarly irritable in both mind and body ! Occurring under such circumstances, we are able in many cases to find nothing more rational to which it should be attributed than idiosyncrasy. Labor pains, although the result of a natural function, have the reputation of being concerned in the production of the complaint before us. Characterized with great severity, in a constitution naturally irritable, labor pains, we have no doubt, are at times the principal cause of the mischief. As yet we have witnessed no case that we knew was due entirely to this cause. Other disturbances we have seen, which renders the matter in our estimation very probable. Not long since, my partner, Dr. Winans, delivered a woman who had very severe labor pains during the last stage of a first labor. As soon as the pains grew severe, she became maniacal, and continued in this condition, perfectly unconscious, until after delivery. In a short time after this event, she returned to her senses, and continued rational. We attended her in her third accouchement. As the pains of labor grew severe, she seemed to lose all command of herself, and had it not been that it was a favorable presentation, and that delivery was speedily accomplished, we have no hesitancy in saying that serious trouble would have occurred.

It is supposed by some that when the pains of labor become severe, too much so for the system to bear, they are superseded by convulsions; convulsions take their place, and they either cease altogether, or become irregular. In James's Burns there is a note published, stating that in a case of parturition attended by Dr. Clark, of London, a convulsion occurred while the hand of the doctor was in the uterus, when of course he had an opportunity of observing how the organ was affected. He remarked that instead of a regular contraction taking place, the uterus seemed to flutter, and be irregularly contracted, and relaxed again quickly; and he was disposed to believe it was in that state during the presence of every case of convulsions. In his work, the Zoonomia, Darwin advances more positively the opinion to which we have alluded. "In difficult parturition," he remarks, "it sometimes happens that general convulsions are excited to relieve the pain of labor, instead of the exertions of those muscles of the abdomen and diaphragm which ought to forward the exclusion of the child. That is, instead of the particular muscular actions which ought to be excited by sensation to remove the offending cause, general convulsions are produced by the power of volition, which still the pain, as in common epilepsy, without removing the cause; and as the parturition is not thus promoted, the convulsions continue till the sensorial power is exhausted—that is, till death." Lately we had a case of labor in which there were no pains except a slight uneasiness in the back. Instead of complaining of uterine pains, the woman complained of her head: it ached very much at times, and at other times it was vertiginous. This state of things continued for some eighteen hours, when uterine pains were developed, after which the distress in the head immediately subsided. During the continuance of the pain and other distress in the head, the os uteri became so much relaxed that when the regular pains of labor set in, it required but a very few of them to produce the expulsion of the child.

Concerning the cause of those convulsions denominated hysterical, there is little definitely known. Of such the symptoms are different from those of the other varieties. Those of a premonitory character are usually absent. The fits are less severe, are not attended with the same amount of cerebral disturbance, and have no frothing at the mouth, nor any of the peculiar sibilating noise so characteristic of the other varieties. Delicate and nervous women, Dewees tells us, are most frequently the subjects of this species of the complaint. As a matter of course, the trouble must be sought for in an investigation of the habit, predisposition, and actual state of the patient affected. Carefully invoked, these in a majority of instances will reveal a pathological foundation, upon which a rational plan of treatment may be predicated.

To sum up our remarks on this head, we submit the following conclusions:

1. The complaint is sometimes the result of primary disease in the brain, as when it proceeds from violent mental emotions, from headache and other cerebral distress during pregnancy, or from epilepsy and other circumstances in early life predisposing the patient to cerebral hyperemia.

2. Most frequently it seems to be developed at the time when the uterus and abdominal muscles are at their greatest state of distension; and hence it is right to regard these conditions as standing, in many instances, in a very close relation to the cause of the complaint.

3. It may depend on irritation of the uterus, from a dead fœtus.

4. It may be caused by overloading or irritating the alimentary canal with indigestible food.

5. Some cases appear to depend on great exhaustion from hemorrhage.

6. It may depend upon habitual constipation;

7. Upon a peculiarly irritable condition of the nervous system of the uterus;

8. Upon violent labor pains.

Upon the above we look as the principal elements of the trouble that exists in connection with the origin of the malady before us. That any one of them may be capable of stirring up those disturbances that characterise the complaint, is not at all unreasonable. Oftener, perhaps, it will be found to be the result of several of them combined. And always the practitioner, in trying to ascertain which of them has had most concern in giving rise to the complaint, will find himself more or less embarrassed. To do this, nevertheless, difficult as it is, is in many instances indispensable to the successful application of remedies.

Post Mortem Appearances.—In regard to the structural lesions pertaining to this disease, our knowledge is limited. There are nevertheless some observations, which, if posted up, may be of some service to the profession. Denman tells us of a case, related to him by Mr. Hewson, where an effusion of blood was found after death on the surface of the brain. He also mentions a case, related by Dr. Hooper, where a coagulum of blood weighing nearly four ounces was found between the dura and pia mater. He himself in no instance ever witnessed an effusion of blood in the brain, though he has seen the vessels very turgid. Of the four cases examined by Dr. Ramsbotham, one showed blood to be extravasated between the dura and pia mater and on the orbital process under the right lobe. In seven of the fifty-four cases contained in the tabular statement of Dr. Lee, a post mortem examination was made. One presented turgescence of the bloodvessels of the pia mater. In two of the others, the vessels of the brain generally were distended. In the fourth, there were softening of the brain, effusion of serum into the ventricles, and tubercles in the lungs. The fifth presented great vascularity around the tuber annulare, with a tablespoonful of serum at the base of the brain. In the sixth, there were serum in the ventricles, a small scrofulous tumor on the basilary artery, and a portion of the right lobe was soft and of a yellowish color. The seventh presented a thick layer of lymph on the surfaces of both hemispheres, the ventricles filled with serum, softening of the base, and veins filled with firm coagula. Dewees gives a case in which there was post mortem dissection: "The longitudinal sinus of the dura mater contained by estimate between two and three ounces of blood; the posterior left ventricle was filled with a bloody serum; the other ventricles appeared sound, as did the other parts of the brain; no other part examined." In addition to the lesions of the nervous centres, there have been some other appearances observed that are worth noticing. Denman says that he has examined many women who have died of convulsions, and in all the heart was unusually flaccid, without a single drop of blood in the auricles or ventricles. He also states in a note, that in almost every case that he has seen, there was, after delivery, a greater or less degree of abdominal inflammation.

Prognosis.—That the term dangerous applies to the complaint before us with considerable relevancy, is a position which the experienced physician will always sanction. Varied, however, it is by the variety with which the patient is affected, and also by a number of other circumstances. As a general rule, it is presumable that the lesions in the hysterical are less grave than in the other varieties; still when even this variety is associated with symptoms of general prostration or hemorrhage, it is by no means free from danger. With the apoplectic variety, which of itself is always exceedingly portending, are occasionally associated some symptoms that are of very serious import. Ushered in with pain in the stomach, this variety, in the estimation of Denman, is apt to be more fatal than where the head alone is affected. The most of the cases we have witnessed attended with this complication terminated fatally. There is something in the manner in which the fits succeed each other, that exercises an influence in the prognosis. The danger, we are told by Denman, is not increased by their frequent return so much as by their increasing violence. This is in accordance with our own observations. In two of our cases that recovered, the number of paroxysms greatly exceeded those that died. Something is thought also to depend on the manner in which patients are attacked. Suddenly invaded, the patient is in more danger than where the disease consumes considerable time in the period of incubation. Dr. Dewees tells us that the most suddenly fatal case he ever saw had no symptons of premonition; without giving any warning, the patient cried out, "My head! my head!" and in a few hours expired. In one of the cases we have reported, the woman first commenced complaining of her head and stomach, and in a few hours afterwards died.

Mortality.—Incident to this affection, there is a degree of mortality which, in spite of all the resources of our art, is high. Of the seven cases we have reported, three terminated fatally, and another one was reduced to a state of confirmed idiotism. Of the eight reported by Dewees, two died; Lee reports fifty-four cases, eighteen of which died; Collins thirty, five of which died; Merriman forty-eight, eleven of which died; Ramsbotham twenty-six, ten of which died; Ingleby thirty-five, eleven of which died; Mauriceau forty-two, nineteen of which died. Thus of two hundred and fifty cases in all, seventy-nine, or nearly one-third, went to a fatal issue. A disease that destroys one in three or nearly so of all its victims, must be regarded as being of the deepest solicitude to every one having anything to do with the duties of an accoucheur Besides the direct mortality, it is quite likely there has been a number of the cases computed to have recovered that were left in a state of infirmity of some kind or other for life.

Treatment.—Considered with respect either to its difficulty or the trouble there often is in making a satisfactory diagnosis, the treatment of the complaint of which we have been speaking has been and will doubtless continue to be among those things in which physicians feel considerably embarrassed. In the absence of a perfect unanimity of sentiment among teachers touching the proper plan of treating any malady, the mass of the profession are thrown upon their own resources, and each one is apt to regard his own individual experience as being the most worthy of confidence. To some extent, this has been the case with reference to the treatment of the disease we are considering. Nearly the same remedies are recommended by all the systematic writers to whom we have had access. With respect, however, to their relative importance, the extent to which their employment should be carried, and the conditions of the system most proper for their use, there seem to be different opinions. That all cases of this or any other complaint require the same remedies, is a position too gross to be the subject of a grave criticism. When the causes giving rise to the complaint, and other circumstances connected with the constitution, habits, etc. of the patient differ, of course we must look for a corresponding difference in the means of relief.

To the remedies which have the most reputation in the treatment of convulsions we will give a brief special consideration.

*Bloodletting* has by all authors had a prominent place assigned it as a remedy. A number urge its value with decided enthusiasm. " We must not" says Burns, " spare the lancet." He concludes all agree on this, and that " there is more danger in taking too little than too much blood." Denman regards it as " the first and most obvious remedy," and refers to his own experience and that of others, showing the necessity, at times, of very copious sanguineous evacuations. Dewees and Gooch also look upon bleeding as the sheet-anchor of the practitioner. That we are able to do more with the lancet than with any other single remedy, or perhaps with all others combined, is a position the truth of which will not be questioned at our hands. But that it is to be indiscriminately used, or that it is the remedy in all cases, is to us not in accordance with reason or experience. While, therefore, we regard it as being valuable in almost all cases, and as being in others that alone by which life is saved, we think that its employment should always be regulated by circumstances—the constitution of the patient, the violence of the convulsions, the effects produced by the loss of blood, the cause giving rise to the malady, etc.

All females affected with the complaint before us are not alike as it regards their strength of constitution and the amount of blood their systems contain. In the first case reported in this paper, the woman was of strong constitution, had a large frame, had her muscular system well developed, and although she was forty-two years of age, her mode of living had done much towards hardening her physical powers and promoting general health. The convulsions in her case were very severe, and attended with an urgent set of symptoms. This case, taken altogether, was properly characterized for copious evacuations of blood. As a consequence, this woman lost in all between eighty and ninety ounces in the course of twenty-four hours, and, as it appeared, with the happiest effects. After the last bleeding, the fits subsided, and then she was delivered of a dead child, and had a rapid convalescence. The third case was that of a young female, who had been in the enjoyment of vigorous, robust health. She was of rather small stature, but inclined to corpulency, and in the sixth or seventh month of utero-gestation. Her convulsions were of the epileptic variety, and at each return they seemed to increase in violence. This is another case in which bleeding was indispensable—was the most powerful means at the command of the practitioner for preventing fatal lesions of the brain or preserving the life of the child. This woman was bled three times, and lost in all some thirty-five or forty ounces of blood, and although delivery by art was resorted to as necessary and proper under the circumstances, still I have no doubt that the woman's life was saved by the amount of blood she lost. These two cases exhibit the conditions of the system in which extensive depletion displays its best effects. Women of large stature, strong constitution, and plethoric habits, are indeed the only ones in which such extensive depletion as was practised in our first case can be made with impunity. Relied upon in such cases, either as a preventive of convulsions, or for the relief of the brain, or for the purpose of relaxing the os uteri, it will display effects of which perhaps no other remedy with which we are acquainted is capable. Here we cordially agree with the remark of Burns, that "there is more danger in taking too little blood than too much." In an opposite state of the physical powers—where, for example, the woman has enjoyed delicate health, has been the subject of some disease which impaired the blood-manufacturing apparatus, or has had extensive hemorrhage—no one would think such extensive depletion either reasonable or efficacious. So feeble is the constitution of some women of small stature and delicate health, that it is impossible to draw much blood from them; and even if it were practicable, eighty or ninety ounces, instead of arresting the disease, might on the contrary destroy life.

Again, no small number of women at the present day are the subjects of dyspepsia, or some fault or other in the blood-manufacturing apparatus. Such labor under various disturbances from the limited quantity, defective quality, etc. of the blood. When these happen to be the subjects of convulsions, it may often be necessary to bleed once, twice, or thrice; but this should be done with great caution, lest a degree of prostration should be induced that might result in fatal consequences. Our fourth case was that of a lady of full powers of constitution. She was attacked with convulsions some twenty days after having been delivered of a healthy child. Afflicted with hemicrania, she was induced to take a dose of patent purgative pills; these produced hypercatharsis; convulsions followed, and she died. This woman was not bled at all. All the symptoms exhibited such a sinking of the powers of life, that bleeding was deemed inadmissible. An opiate was administered, and an epispastic applied to the nape of the neck.

There is another class of cases, which appear to be the result of great debility from hemorrhage. Here is a state of anemia that may either precede or follow delivery. Called to such a case, who would think of opening a vein as a remedy? To stop, indeed, the fatal waste of the vital fluid, and economize and husband the resources of the system, would seem to be most rational, and this appears to be in accordance with what experience we have in these cases. Frictions over the hypogastric region, in order to promote contractions of the uterus, and thereby check the hemorrhage, have been found, we are told, to give prompt relief.

*Local bleeding* may be adopted in some cases—where, for example, the constitution and other conditions of the system forbid general. Delicate women, who have been bled from the arm without relieving the brain, may be benefited by cupping the temples and nape of the neck. Even here, however, strict regard should be had to the strength of the patient, the state of the circulation, etc., in order to avoid reducing the system to that extreme degree of debility from which a recovery could not reasonably be expected.

*Opium* is regarded by most writers and practitioners as being in most cases of questionable propriety. While some reject it altogether, others tell us that under certain circumstances it may be administered with advantage. Unless it is where the convulsions succeed delivery, or are the result of hemorrhage, or are obviously engrafted on a very delicate state of the system, it would probably be best not to administer any of the preparations of opium until the vessels have been emptied by venesection. Bleeding to the extent that the system will bear will not only render the administration of the drug more safe, but it will do much towards increasing its efficacy. Premised in this way by proper abstractions of blood, the administration of opium will in many cases be found to be of service in allaying uterine irritability when excessive, in mitigating the violence of false labor pains,* and in quieting tremors and

---

* In the year 1840, I was called to see a lady in labor, who was the mother of several children. The os uteri was rigid when the first examination was made, and resisted very much the process of dilatation. After it was somewhat opened, the head passed sufficiently to become impacted, and continued in this situation for some ten or twelve hours. During this time, the pains of labor were very severe, and the woman begged urgently for relief. Having been instructed to believe that opium under such circumstances would relieve or mitigate the severity of the pains, I commenced its administration in the form of laudanum of ordinary strength. The first dose produced no effect whatever, and I repeated it until in a spasms. What the value of the article is in cases connected with hemorrhage, we have had no means of ascertaining; but from the fact that it has been found to be of service in hemorrhage connected with other pathological states of the system, we should think that in judicious hands it might be beneficial. There are some cases in which opium is inadmissible, and is calculated to do great injury. We allude to those in which the fits are evidently increasing in violence at each successive paroxysm, or are attended with profound coma, stertorous breathing, and other phenomena indicative of great cerebral congestion.

*Emetics.* When speaking of the etiology of this malady, we noticed that it was once in a while connected with gastric irritation from improper food, etc. Here it would seem that emetics might prove serviceable. In our second case, gastric irritation was present as the result of having eaten unripe fruit. Complaining of great pain in the stomach, the patient took an emetic in doses sufficient to empty the stomach. Although this measure did not arrest the fits, we have no doubt that by its influence they were considerably mitigated. Denman says: "I have not known any woman, who had frequent vomitings in the time of labor, fall into convulsions; nor do these often happen in difficult labors. But women will frequently have vomitings after they have been seized with convulsions, which afford some relief." Construed in the sense in which the author most likely intended, I suppose we are to understand the above remarks as being designed to suggest the importance of this class of articles, both in preventing and mitigating the fits. Capable of accomplishing either of these objects, any article should be entitled to consideration. Prevention, we all know, is better than cure, and mitigation, when nothing more can be accomplished, is always desirable. Discrimination, neverthe-

---

period of ten hours I had administered half an ounce. The pains still continued to recur with regularity and with their former violence. From this experiment and some others that I have made, I conclude that opium possesses very little control over the true pains of labor. False pains it may arrest, and to these its usefulness, I suppose, is pretty much confined.

less, in reference to the cases in which emetics are used is indispensable. No physician would think of giving them in weak, relaxed conditions of the system, or where the convulsions are connected with hemorrhage, or under such circumstances as attended our fourth case, where the disease was apparently the result of the imprudent use of patent cathartic pills.

*Cathartics* no author omits to mention, and perhaps there is no practitioner experienced in the treatment of this disease, who has not witnessed indications requiring their employment, and, at times, decided utility as the result of their administration. As a general rule, the bowels, if not torpid at the commencement, generally become so when the cerebral disturbance is grave; in some instances it is impossible to move them with anything that can be used, until the fits are moderated or altogether checked. Still it is proper after venesection and other means have failed to procure relief, and where the bowels have not been moved during the progress of the complaint, to make a vigorous effort for the accomplishment of this object. Owing to the difficulty of getting patients to swallow while in the insensible state incident to the disease, it is necessary to use some article a small quantity of which if swallowed will answer the purpose. If not prompt in its effects, the cathartic should be invited to action by an enema.

We might go on to enumerate other remedies of which favorable mention has been made in particular cases—such, for example, as the warm bath, dashing cold water in the face, tickling the nostrils, camphor, etc.; but we have already bordered on prolixity, and shall therefore bring this part of our subject to a close, by merely suggesting the utility of counter-irritation in cases where other means have failed to remove the coma and restore the patient to a state of consciousness.

We now proceed to another element in the treatment of convulsions.

Delivery by Art.—In nothing pertaining to the practice of midwifery is the accoucheur more frequently puzzled than in deciding in his own mind upon the circumstances proper for delivery by art. After he has used the lancet and its auxiliaries to the extent that he is warranted in doing, he still in some cases finds the convulsions continuing and perhaps increasing in violence. He knows that if this state of things continue, the issue must be disastrous—that both mother and child must inevitably perish;

> "With remorseless cruelty—
> Spoiled at once, both fruit and tree.
> The hapless babe before his birth
> Had burial, yet not laid in earth."

On the other hand, he knows that many mothers, but more children, have died while attempts were being made at delivery, or the mother afterwards, from the continuance of the convulsions. Embarrassed by such events, the practitioner is too often mortified with his inability to decide upon the proper course to be pursued. Notwithstanding these things, there must be a course which, however imperfect it may be, must be founded on the general experience of the profession in such cases, and the practitioner's own knowledge of the circumstances with which he is surrounded. In order to elaborate this matter a little, we will propose a question or two for consideration:

1. Is it proper, in cases of the sixth, seventh or eighth month of pregnancy, to attempt artificial delivery until there is evidence of the death of the child? In favor of the affirmative of this question no one perhaps can be found. It would be making a sacrifice of the child for at best an uncertainty, that of being able by such a course to save the life of the mother; for we know that it frequently happens that mothers die after delivery as well as before. It is true that in many instances the mother's chance of living would in all probability be increased by emptying the uterus of its contents. With nothing before us, however, but a probability that such would be the case, no individual would be justifiable in proceeding irrespective of a due regard to the life of the child. In cases of the character we are considering, the os uteri is either entirely closed up or in process of dilatation. In the former condition, of course nothing can be done without sacrificing due respect to the child, nor without some considerable hazard to the mother. The exertion, indeed, necessary to dilate the os uteri must subject the woman to much additional suffering, and if the requisite violence which some cases require in order to produce dilatation be used, there could be but little prospect entertained of a recovery. When the parts are in progress of dilatation, interference is generally unnecessary, for a spontaneous expulsion of the fœtus will then most likely be the result. From such considerations, our question must be decided in the negative. Viewed in any or every aspect, this, we conclude, is the proper decision: and it is not more in accordance with our feelings than with what we believe to be the most enlightened system of practice.

Our next question is—

What are the circumstances which make delivery by art proper and justifiable? In reply to this, it may be stated—

1st. Where there is evidence, in a case of premature labor, that the child is dead and the os uteri open or easily dilatable, there is no propriety in waiting for the spontaneous expulsion of the fœtus. Whatever its agency may have been, while alive, in originating or keeping up the convulsions, one thing is obvious, that after its death, operating as a foreign body in the uterus, and imparting to the circulation of the mother a sudden change, there can be no propriety in permitting it to remain, but, on the other hand, many good reasons why its removal may put a stop to the fits.

2d. In cases of convulsions that take place at or about the full period of pregnancy, prompt delivery, when the parts are dilated or capable of being dilated, is one of the measures of relieving the fits upon which considerable reliance may be placed. When this is accomplished, it very often happens that the fits cease altogether. Besides the relief which usually accrues to the mother from this measure, it may be presumed that it often saves the life of the child, by removing it from a situation in which it must have perished. Hence when convulsions happen to women who have gone the full period of gestation, it is no uncommon thing to save the child by delivery, when we are unable to save the mother. This was done in our fifth case.* Here the parts were in a favorable condition, and the woman had gone to full time; the forceps were applied when the tendency to convulsions was first noticed. Before, however, the child was delivered, the convulsion had made its advent. The woman died in the second convulsion, but the child was saved.

In concluding our remarks on the treatment of this malady, we submit the following positions:

1. Each case of the disease is likely to have some peculiarity, and therefore requires discrimination in regard to the selection of remedies.

2. Bloodletting is not only the most general remedy, applicable to almost all cases, but it is also the most valuable.

3. The extent to which depletion should be carried will depend not so much on the violence of the convulsions as upon the habit of body, strength of constitution, etc., of the patient.

4. Opium, emetics and cathartics have considerable value as auxiliaries.

5. Delivery by art is a measure of great value, but should never be performed without strict regard to both mother and child.

Jamestown, Ohio, February 10, 1847.

---

* Dr. Winans introduced the forceps and delivered the child in this case. It happened that the fit came on before he had succeeded in applying the instrument, at which time he had an opportunity of observing the effect which it produced on the uterus. Instead of the regular contractions, the organ, he observed, fluttered around his hand, and had imperfect irregular contractions.

